# Supplementary material for: Mitochondria Clearance Enables Macrophage-Driven Maturation of iPSC-Derived Cardiomyocyte Metabolism
Source: Cell Mol Bioeng. 2026 Jun 3;19(3):359–73. doi: 10.1007/s12195-026-00915-z (PMC13365287; doi:10.1007/s12195-026-00915-z)
Supplement: Supplementary file 1 — Supplementary file1 (PDF 296 kb) [file 12195_2026_915_MOESM1_ESM.pdf]

Supplemental Figures

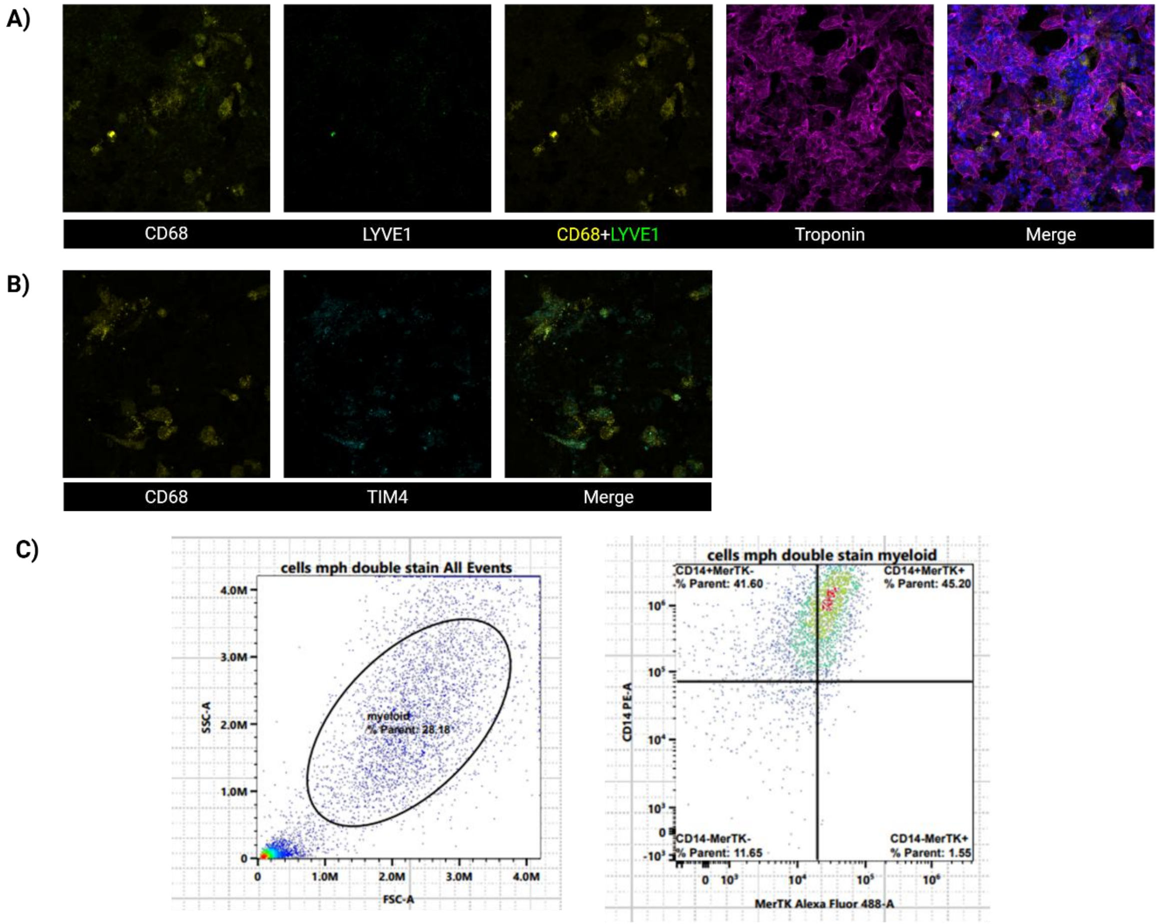

Supplemental Figure 1: iPSC-derived macrophages take on cardiac resident phenotype in culture with developing cardiomyocytes. A) immunostaining of iMϕ after 3 days culture with d9 iCMs. Mϕ intercalate with iCM troponin and express some LYVE1. B) immunostaining of iMϕ showing expression of TIM4 by CD68-expressing macrophages. C) flow cytometry plot showing freshly harvested macrophage expression of CD14 and MerTK.

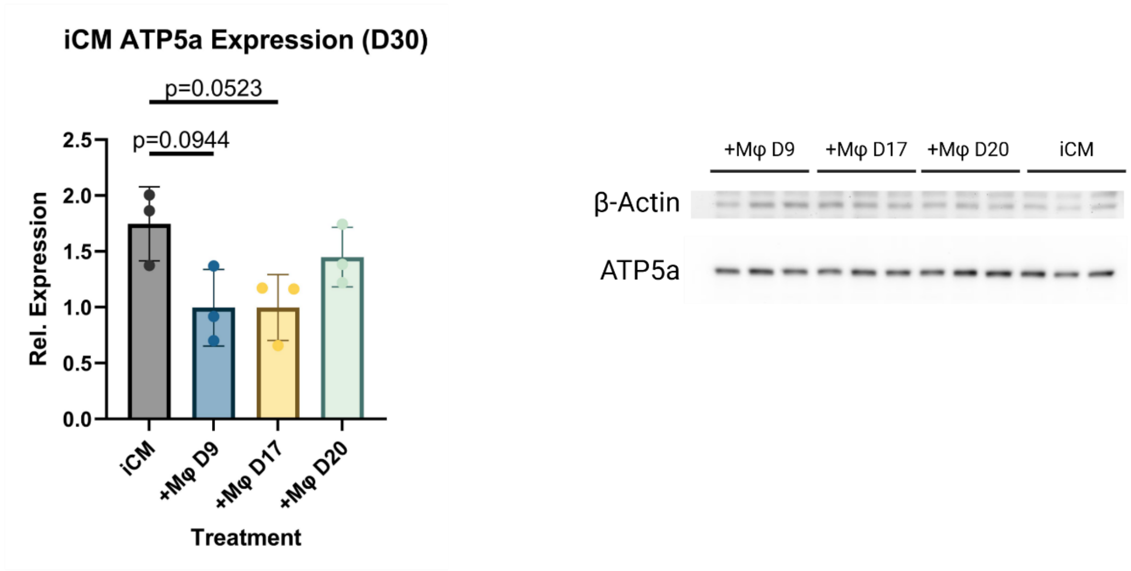

Supplemental Figure 2: Expression of ATP Synthase and Parkin in iCMs after Coculture with Macrophages. Blot of d30 iCMs with or without coculture with macrophages on day 9, 17, or 20. Protein expression is calculated relative to expression of β-actin. P values from Welch's t-test.

Supplemental Methods

Flow Cytometry

Suspended macrophage precursors were collected freshly from embryoid body cultures and centrifuged. Pellets of 1 million cells were resuspended in flow cytometry (FC) buffer (phosphate buffered saline (Corning) with 0.5% w/v bovine serum albumin (Thermo)) supplemented with 100µg/ml human IgG (Invitrogen) and blocked on ice for 30 minutes. Tagged primary antibodies against CD14 (clone 61D3, phycoerythrin, eBioscience) and MerTK (clone HMER5DS, Alexa Fluor 488, eBioscience) were added directly to blocking solution and incubated for 30 minutes on ice in the dark. Cells were then washed three times in PBS and data was acquired using a Cytek Northern Lights (NL)-CLC flow cytometer.

Western Blotting

Cells were lysed using RIPA buffer supplemented with protease inhibitor cocktail. The protein concentration of each sample was quantified via bicinchoninic acid (BCA) assay (Thermo Fischer), and equal amounts of protein were separated by 10 or 12% sodium dodecyl sulfate-polyacrylamide gel electrophoresis (SDS-PAGE) at 200 V for 45 min and transferred to the polyvinylidene difluoride (PVDF) blotting membrane at 100 V for 1 hour. The PVDF membranes were blocked in SuperBlock Blocking Buffer (37536; Thermo Fisher Scientific) for 1 hour at room temperature and incubated at 4°C overnight with primary antibodies. After three Tris-buffered saline with Tween20 washes for 10 min each, the membranes were incubated with goat HRP-conjugated anti-rabbit or mouse secondary antibodies (ab205718, Abcam, 1:4000) for 1 hour at room temperature. Membranes were then incubated with a chemiluminescent substrate (Clarity ECL, Bio-Rad) for 3 minutes and imaged on a ChemiDoc-It2 system (UVP, Analytik Jena) using VisionWorks software and the pixel density of each protein band was quantified using ImageJ. Blots were stripped using Restore Western Blot Stripping Buffer (21059; Thermo Fisher Scientific) and reprobed to visualize multiple proteins.
